# The rise of the throat distemper

**DOI:** 10.12669/pjms.40.2(ICON).8954

**Published:** 2024-01

**Authors:** Aneela Hussain

**Affiliations:** 1 Dr. Aneela Hussain, FCPS Medicine, FCPS Infectious Diseases. Consultant, The Indus Hospital, Karachi, Pakistan. Email: doc.aneela@yahoo.com

**Keywords:** Diphtheria, Vaccination, Throat

## Abstract

Diphtheria vaccination in the EPI program has controlled much of the childhood infection. Nevertheless, sporadic adult cases of Diphtheria come up every now and then in Pakistan and other South-Asian countries. This is, most likely, due to the lack of booster dosing of Diphtheria vaccine in adulthood. In an effort to suppress the spread of this infection, adult vaccinations need to be mandated.

The resurgence of a largely forgotten, old world malady, Diphtheria. First described by Hippocrates in the 5^th^ century BC, as causing havoc and taking many lives. It is caused by a bacteria called Corynebacterium diphtheria, which was named after the Greek word for “Leather” on account of its propensity to form a thick, adherent, grey membrane in the throat of the person infected, literally choking them to death.^1^ Being a highly contagious, droplet infection, it spreads easily in close contacts; even to healthcare workers during the first few minutes of patient examination.^2^ Not only that, carriers of diphtheria are also likely to spread the disease.^2^,^3^

Diphtheria, is exotoxin-mediated and starts like any upper respiratory tract infection with fever, sore throat, sputum production, and later turns into an obstructive airway disease requiring tracheostomy. It can lead to toxic cardiomyopathies and neuropathies.^2^ The diagnosis has to be a clinical one for prompt antibiotics and Diphtheria antitoxin administration, the latter being a life-saving measure as it neutralizes the Diphtheria-toxin. Throat swabs are needed for Gram stain and cultures for a more objective confirmatory diagnosis.^3^,^4^

Diphtheria does not build one’s natural immunity, and re-infections can occur.^4^ Curbing the infection, therefore, requires giving a booster dose at four to six years of age and also strictly enforcing adult booster doses every 10 years, as suggested by WHO and CDC, in the form of a combination vaccine with Tetanus toxoid.^4^ We can further restrict the spread of infection by ensuring antibiotic prophylaxis in all contacts of patients.^2^

However, outbreaks have been reported in our neighboring countries including many states of India and Bangladesh, and 46 cases have been reported from different cities of Pakistan, just last year, mainly in Khyber Pakhtunkhwa. Likewise, around 169 cases were seen by Pakistan in the last 20 years. Noteworthy here is the emergence of adult cases diagnosed with Diphtheria.^3^,^5^ Diphtheria being misdiagnosed as any ordinary upper respiratory infection has been found to be the number one cause of mortality in both India and Pakistan which may be due to lack of proper examination and awareness in the medical personnel.^2^ In 2022 alone, 45 diphtheria related deaths were recorded- alarming indeed, given the easily preventable nature of the infection.^6^

Diphtheria vaccination is a part of the EPI program, till 14 weeks-of-age; and thereafter, protects children well through their early teenage years. However, adults, due to a waning antibody response to their childhood immunization, are prone to getting infection and this diminishing response to antibodies is a big contributing factor in causing this age-shift of Diphtheria infections. Around 90% adults do not have protective antibody levels despite completing diphtheria vaccination in childhood.^7^-^9^ On top of that, failing to get booster doses, later on, due to resources or lack of availability leads to infection in adolescents and adults.^2^-^4^,^10^,^11^

A Pakistani sero-epidemiological survey which found a drop in levels of Diphtheria antibody gradually after 10 years of age and further after 30 years of age has drawn attention to the need for adult booster dosing.^12^ It has been found that after 20 years-of-age, the childhood vaccination is only 63% effective.^2^ As antibiotics alone are insufficient to treat the infection, Diphtheria anti-toxin availability in hospitals has to be mandated, across the country.^3^ It is a well-known fact that the anti-toxin, when administered with 24 hours after the diagnosis, decreases mortality by 76%.^2^

The ongoing socioeconomic upheaval after COVID-19, recent floods, and inflation, has added to the financial crisis of the country, leading to further decline in healthcare services. Mass Td booster campaigns, making Diphtheria anti-toxin available in hospitals and guaranteeing prophylaxis in contacts, are the only means to ending the sporadic outbreaks and controlling this mortal infection.
